# Phase II Study of Gemcitabine, Peg-Asparaginase, Dexamethasone and Methotrexate Regimen for Newly Diagnosed Extranodal Natural Killer/T-Cell Lymphoma, Nasal Type: Final Analysis With Long-Term Follow-Up and Rational Research for the Combination

**DOI:** 10.3389/fonc.2022.796738

**Published:** 2022-01-24

**Authors:** Yu Wang, Cai-Qin Wang, Peng Sun, Pan-Pan Liu, Hang Yang, Han-Yu Wang, Hui-Lan Rao, Su Li, Wen-Qi Jiang, Jia-Jia Huang, Zhi-Ming Li

**Affiliations:** ^1^ Department of Medical Oncology, Sun Yat-sen University Cancer Center, Guangzhou, China; ^2^ State Key Laboratory of Oncology, South China, Collaborative Innovation Center for Cancer Medicine, Guangzhou, China; ^3^ Department of Medical Oncology, The Sixth Affiliated Hospital of Sun Yat-Sen University, Guangzhou, China; ^4^ Department of Radiation Oncology, Sun Yat-sen University Cancer Center, Guangzhou, China; ^5^ Department of Pathology, Sun Yat-sen University Cancer Center, Guangzhou, China; ^6^ Department of Clinical Research, Sun Yat-sen University Cancer Center, Guangzhou, China

**Keywords:** extranodal NK/T cell lymphoma, high-dose MTX and gemcitabine, long-term follow-up, final analysis, synergistic effect

## Abstract

Patients with extranodal natural killer/T-cell lymphoma (ENKTL), nasal type are benefit from peg-asparaginase, gemcitabine, and methotrexate. Therefore, we conducted a prospective phase II trial using a combination of these drugs as GAD-M regimen in naïve ENKTL patients, simultaneously, explored the combinational mechanism. The GAD-M regimen was administered for 6 cycles sandwiched by radiotherapy for stage I/II and 6 cycles for stage III/IV patients. After 6 cycles, the overall response rate of 36 patients was 91.6%, and the complete remission rate increased to 83.3%. The 3-year progression-free survival (PFS) and overall survival (OS) rates were 74.8% and 77.8%, respectively. The 5-year PFS and OS were 68.3% and 77.8%. No patient suffered from the central nervous system (CNS) relapse. Most patients experienced recoverable liver dysfunction and anemia in this study. The plasma MTX concentration ratio at 12 to 24 hr during the first cycle could be an early predictor of outcomes in ENKTL (PFS, *P*=0.005; OS, *P*=0.002). Additionally, we found that high dose MTX (HD-MTX) and gemcitabine had the synergistic effect of ENKTL cell *in vitro*. Mechanistically, we demonstrated that the combination could lead to obviously apoptosis in ENKTL cell with extremely release of reactive oxygen spices (ROS), which mediated by endoplasmic reticulum stress. In conclusion, the GAD-M regimen could be a new choice to newly diagnosed ENKTL, especially for stage I/II patients. Furthermore, our results showed the synergy effect of HD-MTX with gemcitabine in ENKTL.

**Clinical Trial Registration:**

This trial was registered at www.clinicaltrials.gov as #NCT01991158.

## Introduction

Extranodal NK/T-cell lymphoma (ENKTL) is a highly aggressive lymphoma that has a geographic predilection in Asian and South American populations (1–4). In China, the incidence of ENKTL is higher and secondary to the diffuse large B-cell lymphoma, accounting for approximately 11% of all lymphomas (5). Almost 75% of ENKTL cases typically involve the nasal cavities, with the common initial symptoms being nasal obstruction, nasal discharge, and epistaxis; caused by the nasal lesions (6, 7). They can also develop rapidly in any other site such as the nasopharynx, skin, orbits, gastrointestinal tract, testis, and lymph nodes.

It has been shown that cyclophosphamide, doxorubicin, vincristine, and prednisone (CHOP) chemotherapy or CHOP-like regimens were associated with poor outcomes in ENKTL (8) because the multidrug resistance (MDR) P-glycoprotein expressed by ENKTL cells acts as an efflux pump to combat the effects of various drugs, including cyclophosphamide and doxorubicin (9–11). As such, regimens incorporating anthracycline-containing chemotherapy have been abandoned as treatments for ENKTL (1, 2, 8). Instead, a series of regimens incorporating peg-asparaginase/L-asparaginase-based chemotherapy have shown greater efficacy (12–18). In China, the peg-asparaginase/L-asparaginase, gemcitabine, and oxaliplatin (PGEMOX/GELOX) regimen has been widely used (17, 18). However, the optimal treatment for ENKTL has not yet been fully defined, and the dilemma regarding which drugs should be combined with peg-asparaginase/L-asparaginase continues to evolve.

Methotrexate (MTX) is another important drug in ENKTL therapies and is contained in both the dexamethasone, MTX, ifosfamide, L-asparaginase, and etoposide (SMILE) and asparaginase, MTX, dexamethasone (AspaMetDex) regimens used for the treatment of ENKTL (12, 13, 16). The PGEMOX/GELOX regimen does not contain MTX. We therefore conducted this clinical trial using the chemotherapy regimen gemcitabine, peg-asparaginase, dexamethasone, and MTX (GAD-M) to treat newly diagnosed ENKTL, and involved-field radiotherapy (IFRT) was also added to the treatment of stage I/II ENKTL. The objective of this present study was to investigate the efficacy and safety of the GAD-M regimen in newly diagnosed ENKTL patients. Furthermore, we applied a series of rational research methods to investigate how the GAD-M regimen produced a synergic efficacy in ENKTL patients. This trial is registered at www.clinicaltrials.gov as #NCT01991158.

## Patients and Methods

### Patients

Newly diagnosed ENKTL patients were eligible for this study if they met the following inclusion criteria: (a) a histologically confirmed diagnosis of ENKTL according to the WHO classification (19), (b) no previous anti-cancer treatment (chemotherapy, radiotherapy, targeted therapy or stem cell transplantation), (c) age: 18-80 years old, (d) Eastern Cooperative Oncology Group (ECOG) performance status: 0-3, (e) life expectancy > 3 months, (f) clinical stage according to Ann Arbor staging system: I-IV, and (g) adequate hematological, liver and renal function (i.e., WBC count ≥ 4×10^9^/l, hemoglobin ≥ 100 g/l, platelet count ≥ 90×10^9^/l, bilirubin <1.5×ULN, alanine transaminase (ALT) or aspartate aminotransferase (AST) < 2.5×ULN and serum creatinine < 1.5×ULN), normal coagulation and cardiac functions. Patients were excluded if they: were pregnant or breastfeeding, had other concomitant malignant tumor(s), severe infection, and any coexisting medical problems, such as congestive heart failure, liver cirrhosis, active hemorrhage and so on, that could cause poor compliance with the study protocol.

All patients signed an informed consent form before enrolment. This study adhered to the norms of the Helsinki Declaration and Good Clinical Practice Guidelines. The study was approved by the ethics committee of the Sun Yat-sen University Cancer Center.

### Treatment and Protocol Design

This is a prospective single-arm, open-label phase II study. The patients received six cycles of the GAD-M regimen. The regimen was given every 3 weeks. The GAD-M dosage and administration schedule were as follows: gemcitabine: 1000 mg/m^2^ *via* intravenous drip on days 1 and 8, peg-asparaginase: 2500 U/m^2^ *via* intramuscular injection on day 1, dexamethasone: 20 mg *via* intravenous drip on days 1-3, and MTX: 3000 mg/m^2^ *via* a continuous intravenous drip for 12 hr on day 1. All of the patients in our study received leucovorin rescue and alkaline hydration for 3 days during and after the treatment with high-dose MTX. Their MTX plasma level was monitored during treatment (0, 12, 24, 36, and 48 hr from the beginning of continuous treatment with intravenous MTX).

Stage I/II ENKTL patients received 6 cycles of the GAD-M regimen sandwiched by IFRT after the 2^ed^-4^th^ cycle. Three-dimensional (3D) conformal radiation therapy was given to stage I/II ENKTL patients using 4 or 6 megavoltage (MV) photons generated from a linear accelerator. The IFRT dose was 50-55 greys (Gy) in 25 fractions applied at 2 Gy per day, 5 fractions per week. For patients in stage III/IV, the GAD-M regimens were repeated for 6 cycles without IFRT.

### Response and Safety Assessments

The primary endpoint was the overall response rate (ORR) after six cycles of the GAD-M regimen. The secondary endpoints were complete response rate (CRR), 3-year progression-free survival (PFS), 3-year overall survival (OS), and toxicity.

The tumor response was assessed by computed tomography scan, Magnetic Resonance Imaging (MRI), and/or Positron Emission Tomography (PET) scan and classified as a complete response (CR), partial response (PR), stable disease (SD), or progressive disease (PD) (20). The treatment response was assessed after every 2 cycles of the GAD-M regimen during treatment according to the Revised Response Criteria for malignant lymphoma (20). Follow-up was performed every 3 months in the first 2 years, every 6 months during the next 3 years, and then annually.

All adverse events experienced after chemotherapy were graded according to the Common Terminology Criteria for Adverse Events (CTCAE) version 4.0.

### Cell Culture

NK/T cell lymphoma cell lines NKYS, SNK6 were obtained from the American Type Culture Collection (ATCC). The cells were cultured and maintained in RPMI 1640 medium (Life Technologies) supplemented with 10% fetal bovine serum (FBS), 100 IU/ml IL2, 2mM L-glutamine, 1% penicillin/streptomycin (Life Technologies) at 37°C and 5% CO2.

### CCK8 Assay

NKYS, SNK6 cells were seeded in triplicate in 96-well plates at 1×10^5^ cells per well with 200 μl completed medium for 24 hr. Then, the cells were treated with different concentrations of MTX (1 nM-100 mM) and gemcitabine (0.126 nM-12.6 mM) alone or together. CCK8 solution (Dojindo, Japan) was used according to the manufacturer’s operating instructions, and the IC50 value was calculated using the Prism7 software every 24 hr until 96 hr after treatment.

Synergy in cell viability assays was determined by plotting isobolograms and calculating the combination index (CI) value using the CalcuSyn software. In detail, CI < 1 indicates synergy, CI = 1, additive and CI > 1, antagonism. The results were representative of three individual experiments.

### Determination of Cellular Oxygen Species (ROS)

Cellular ROS production was detected using a 2’,7’-dichlorofluorescein diacetate (DCFH-DA) probe24 (Beyotime, China) according to the manufacturer’s protocol. Briefly, a total of 1.5 × 10^5^/ml cells in 1 ml was seeded in 12-well dishes. The treated cells were washed with PBS twice and incubated with 10 μM of DCFH-DA for 30 min. DCFH-DA fluorescence was measured by a FACS CytExpert 2.0 (Beckman Coulter, USA).

### Flow Cytometry of Apoptosis

Apoptosis was then detected with annexin V-FITC/PI staining kit (Nanjing KeyGen Biotech). NKYS, SNK6 cells were treated with normal conditions, MTX (20 mM), gemcitabine (5 μM), or combination treatment for 24-48 hr, respectively. The cells were then collected, washed, and stained in a working solution (500 µl binding buffer with 5 µl annexin V-FITC and 10 µl PI) for 15 min at room temperature in the dark. The FACS CytExpert 2.0 (Beckman Coulter) was used to detect and analyze apoptotic cells. Annexin V-FITC ^+^ and annexin V-FITC ^+^-PI ^+^ cells were considered as apoptotic cells.

### Western Blot Analysis

Proteins were extracted from cells and then used for western blot analysis. Drug treated cells were lysed with RIPA buffer containing a protease inhibitor cocktail and a phosphatase inhibitor cocktail (CWBIO, USA). Proteins were resolved by SDS-PAGE, transferred to PVDF membranes (Bio-Rad, USA), blocked with 5% skimmed milk powder. Then, the membranes were detected by incubation with 1:1000 dilutions of primary antibodies, washed, and incubated with Goat anti-rabbit-HRP antibodies and developed using WesternBright™ ECL (Advansta, USA). The following primary antibodies were used for western blot analysis: anti-p-IRE1α antibody, anti-IRE1α antibody, anti-CHOP antibody, anti-Cleaved caspase 3, anti-caspase 12, anti-Bcl2 Ser70, anti-p-p38 antibody, anti-p38 antibody (Cell Signaling Technology, USA), anti-β-catenin and anti-GAPDH (Proteintech, USA).

### Statistical Methods

The expected ORR was estimated to be 80% and the threshold ORR was estimated to be 60% based on our hospital history data (21, 22). Our trial was designed to have a statistical power of 80% to test the following one-sided hypothesis regarding the actual probably of an overall response (p); H0: P less than or equal to 60% (60% being the historical response while using conventional therapy) versus H1: P 80% (80% being the expected response), with a type I error of 5%. The number of suitable patients required for our study was calculated to as at least 36.

PFS was defined as the interval between the date of diagnosis and the date of first relapse, progression, death, or last follow-up. OS was measured from the day of diagnosis to death or last follow-up. All data were entered into the SPSS statistical software. The Kaplan-Meier methods and log-rank tests were used to calculate the survival rates and compare survival curves, respectively. Cox regression analysis was used to identify significant predictors of PFS or OS. *P <* 0.05 was considered significant.

Comparisons between two mean values were performed by two-tailed unpaired Student’s *t*-test using the Prism8 software. Significance testing was performed by one-way ANOVA with the Tukey’s posthoc testing for multiple pairwise testing or by parametric or nonparametric Student’s *t*-test as appropriate. *P* < 0.05 was considered significant.

## Results

### Patient Characteristics

A total of 38 patients diagnosed at the Sun Yat-sen University Cancer Center from November 2013 to October 2015 were consecutively considered for this study. Thirty-six patients had at least one evaluable response. Two patients were excluded was one had liver cirrhosis complications that met exclusion criteria, and the other one was already 81 years old when he signed the informed consent. The main clinical characteristics of the 36 patients are summarized in **Table 1**. The median age was 47 years old (range, 18 to 73 years old), and 4 patients (11.1%) were older than 60 years old. The male to female ratio was 25:11. Most of the patients (86.1%) had a favorable Eastern Cooperative Oncology Group Performance Status (ECOG PS: 0-1), and the ECOG PS was no more than 2 in all patients. Twenty-two patients (61.1%) had B symptoms at presentation. Thirty-one patients (86.1%) were classified as Ann Arbor stage I or II, while 5 patients (13.9%) as stage III or IV. Serum lactate dehydrogenase levels were high in 10 patients (27.8%). High Epstein-Barr Virus (EBV) DNA copies (EBV DNA > 10^3^ copies/ml) were present in 22 patients (61.6%), and detectable EBV DNA copies (EBV DNA > 0 copies/ml) were present in 26 patients (72.2%).

**Table 1:** Baseline patient characteristics (n = 36).

Characteristic	Number of assessable patients	(%)
**Age (years)**		
Range (Median)	18-73 (47)	
> 60	4	11.1
≤ 60	32	88.9
**Gender**		
Male	25	69.4
Female	11	30.6
**ECOG PS**		
0-1	31	86.1
2-3	5	13.9
**B symptoms**		
Yes	22	61.1
No	14	38.9
**Ann Arbor stage**		
Stage I-II	31	86.1
Stage III-IV	5	13.9
**LDH**		
> 245 U/l	10	27.8
≤ 245 U/l	26	72.2
**High EBV DNA copy number**		
**>** 10^3^ copies/ml	22	61.1
≤ 10^3^ copies/ml	14	38.9
**Detectable EBV DNA copy**		
> 0 copies/ml	26	72.2
≤ 0 copies/ml	10	27.8
**CRP**		
> 10 mg/l	14	38.9
≤ 10 mg/l	22	61.1
**IPI**		
0	19	52.8
1	12	33.3
≥ 2	5	13.9
**PINK-E**		
Low	25	69.4
Intermediate	4	11.1
High	7	19.4
**KPI**		
0-1	21	58.3
2-4	15	41.7

ECOG PS, Eastern Cooperative Oncology Group performance status; B symptoms consist of unexplained fever above 38℃, night sweating, or weight loss of more than 10% within 6 months; LDH, lactate dehydrogenase; EBV DNA, Epstein-Barr Virus deoxyribonucleic acid; CRP, C-reactive protein; IPI, International Prognostic Index; PINK-E, prognostic index for natural killer lymphoma–Epstein-Barr virus; KPI, Korean Prognostic Index.

### Clinical Response

**Figure 1:**
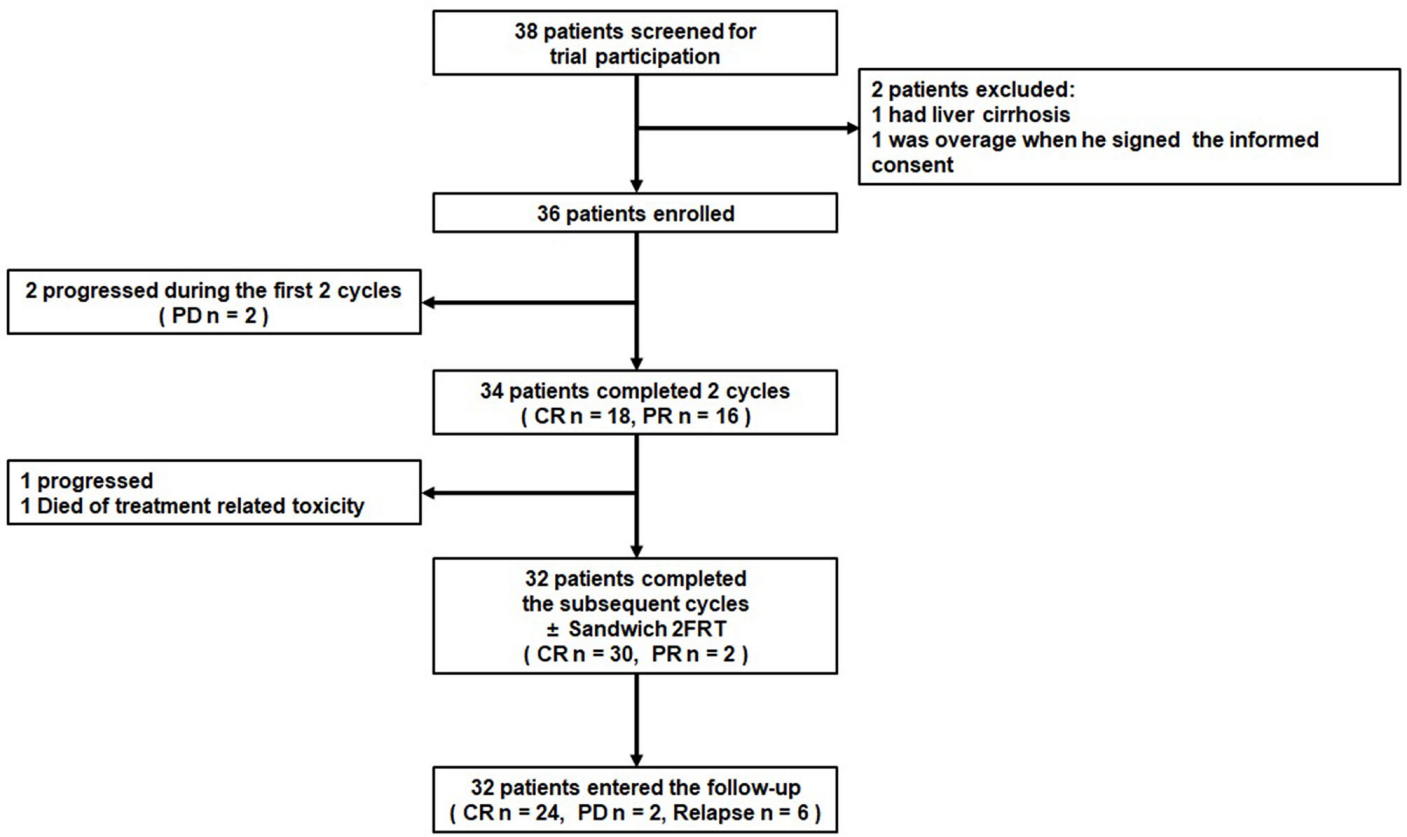
Summary of outcomes in ENKTL treated with the GAD-M regimen.

In all, the 36 patients received 199 cycles of GAD-M regimen chemotherapy. All patients completed at least 2 cycles of the GAD-M regimen and had at least 1 response evaluation. The treatment scheme and outcome are summarized in **Figure 1**. After 2 cycles of GAD-M, the ORR and CRR were 94.4% (34/36, 95% CI: 87.0%-100.0%) and 50.0% (18/36), while the disease rapidly progressed in two patients with stage IV. The ORRs of stage I/II and stage III/IV patients were 100.0% (31/31) and 60.0% (3/5), respectively, while the CRRs were 54.8% (17/31) and 20.0% (1/5), respectively. After 6 cycles of GAD-M, the ORR was still 91.6% (33/36, 95% CI: 82.6% - 100.0%), whereas the CRR had increased to 83.3% (30/36). The ORRs of stage I/II and stage III/IV patients were 100.0% (31/31) and 40.0% (2/5), respectively, and the CRRs increased in both groups to 90.3% (28/31) and 40.0% (2/5), respectively.

### Survival and Relapse

**Figure 2:**
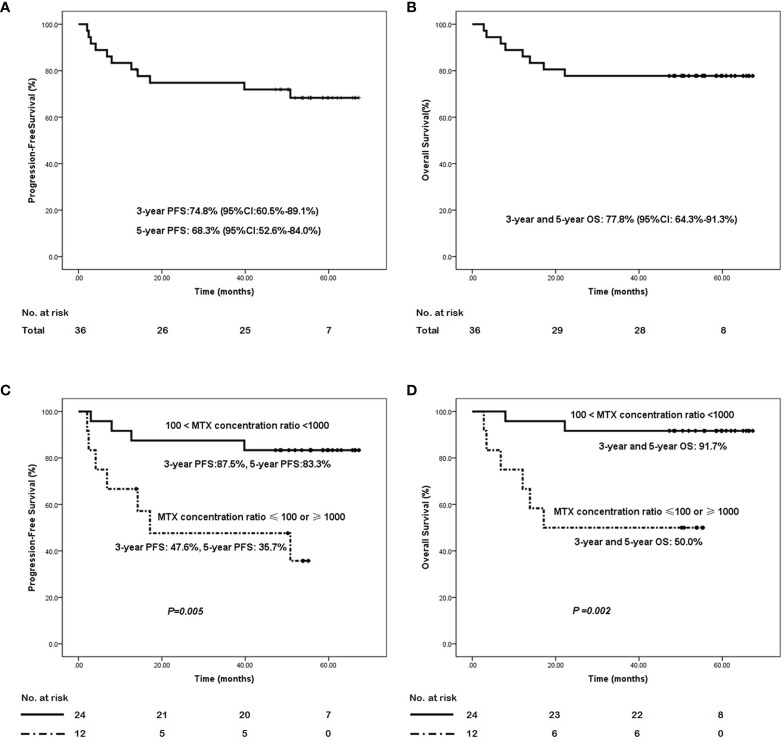
The survival curves of the ENKTL patients with the GAD-M regimen. **(A)** progression-free survival curve and **(B)** overall survival curve in the 36 ENKTL treated with the GAD-M regimen. **(C)** progression-free survival curve and **(D)** overall survival curve in patients according to the plasma MTX concentration ratio from 12 to 24 hr of the first cycle in the treatment with the GAD-M regimen.

The median follow-up for all 36 patients was 54.5 months (range, 5.9-67.3 months). In this study, the median PFS and OS were not reached until the last follow-up; hence, the 3-year PFS and 5-year PFS were 74.8% (95% CI: 60.0%-89.1%) and 68.3% (95% CI: 52.6%-84.0%), respectively. The 3-year and 5-year OS were both 77.8% (95% CI: 64.3%- 91.3%). When analyzed according to Ann Arbor stage, the 3-year PFS rates in stage I/II and III/IV disease were 80.4% (95% CI: 66.3%-94.5%) and 40.0% (95% CI: 0%-82.9%), respectively. The 5-year PFS were 77.0% (95% CI: 62.1%-91.9%) and 20.0% (95% CI: 0%-55.1%), respectively, while the 3-year OS, similar with the 5-year OS, were 80.6% (95% CI: 66.7%-94.5%) and 60.0% (95% CI: 17.1%-100.0%) in stage I/II and III/IV disease, respectively. The PFS and OS survival curves for all the 36 ENKTL patients are shown in **Figures 2A, B**.

In all, eleven out of thirty-six patients (30.6%) who were treated with the GAD-M regimen who completed the follow-up period experienced disease progression after a median of 8.0 months (range, 2.1-50.8 months), and seven of these patients (19.4%) died of lymphoma after a median of 8.0 months (range, 2.8-22.2 months). During chemotherapy, three patients experienced disease progression. All three of them had widely metastasized stage IV disease. Two had progressive disease which rapidly resulted in death. Another experienced disease progression in the spleen after 4 cycles of GAD-M and was treated with peg-asparaginase, dexamethasone, ifosfamide, carboplatin, and etoposide (P-DICE) as a second-line treatment and prednisone, etoposide, procarbazine and cyclophosphamide (PEPC) as salvage chemotherapy. This patient was still alive at the last follow-up. During the follow-up period, five patients with stage II disease experienced disease progression and died of lymphoma. Two other patients with nasopharyngeal posterior wall invasion had disease relapsed in their vocal cords after CR was reported following 6 cycles of the GAD-M regimen sandwiched with IFRT. These two patients received chemotherapy and radiotherapy in turn for salvage treatment, and they were alive at the last follow-up. Another patient in stage IV who had skin lesions and superficial lymph node involvement had disease relapsed in the skin of the cheek in 2019. All other patients remain in follow-up. No patient experienced central nervous system (CNS) relapse in our study.

### Toxicity

The main adverse events of GAD-M are summarized in **Table 2**. The most common grade 3/4 hematological adverse event was anemia (47.2%). Grade 3/4 leukocytopenia and neutropenia were observed in 19.5% and 36.1% of the patients, respectively. Thrombocytopenia was mostly graded as 1/2 (27.8%). The major non-hematological side-effects to GAD-M regimens were hypoalbuminemia (100.0%), increased transaminases (89.0%), and hyperbilirubinemia (52.8%). While grade 1/2 non-hematological toxicities were frequently observed during GAD-M treatment, grade 3/4 toxicities were rare. Ten patients (27.8%) had decreased fibrinogen probably related to peg-asparaginase, and this side-effect was reversible when treated with fresh-frozen plasma and cryoprecipitate. There were no cases of pancreatitis or thrombus in our study. One patient, who was a 61-year-old man, died of treatment-related toxicity due to electrolyte disorders caused by severe vomiting. None of the other patients suffered from this adverse event.

**Table 2:** Toxicity profiles from the GAD-M regimen.

Toxicity	No. of Adverse Events (%)								
	Grade 1		Grade 2	Grade 3			Grade 4	Grade 5	Total
**Hematological AEs**									
Anemia	4 (11.1)	15 (41.7)			17 (47.2)		0	0	36 (100.0)
Leukocytopenia	8 (22.2)	14 (38.9)			6 (16.7)		1 (2.8)	0	29 (80.6)
Neutropenia	8 (22.2)	7 (19.4)			10 (27.8)		3 (8.3)	0	28 (77.8)
Thrombocytopenia	2 (5.6)	8 (22.2)			2 (5.6)		4 (11.1)	0	16 (44.4)
**Non-hematological AEs**									
Hypoalbuminemia	8 (22.2)	26 (72.2)			2 (5.6)		0	0	36 (100.0)
Increased transaminases	24 (66.7)	6 (16.7)			2 (5.6)		0	0	32 (89.0)
Hyperbilirubinemia	9 (25.0)	6 (16.7)			4 (11.1)		0	0	19 (52.8)
Decreased fibrinogen	2 (5.6)	3 (8.3)			5 (13.9)		0	0	10 (27.8)
Mucositis	2 (5.6)	6 (16.7)			0		0	0	8 (22.2)
Vomiting	2 (5.6)	2 (5.6)			0		0	1 (2.8)	5 (13.9)
Increased Cr	3 (8.4)	1 (2.8)			1 (2.8)		0	0	5 (13.9)
Abdominal pain	2 (5.6)	0			0		0	0	2 (5.6)
Epistaxis	1 (2.8)	0			0		0	0	1 (2.8)
Pancreatitis	0	0			0		0	0	0

AE, adverse event; Cr, creatinine.

### The Influence of MTX Plasma Concentrations

The blood MTX levels were measured at 0, 12, 24, 36, and 48 hr from the start of MTX treatment. We analyzed the relationships between the MTX plasma concentration at any time point and patient outcomes. We found that the plasma MTX concentration did not influence the ENKTL outcome at any separate time point. Nevertheless, the changes in MTX plasma concentrations observed over time significantly influenced ENKTL effects and survival. We found that the plasma MTX concentration ratio calculated at 12 to 24 hr(12hs plasma MTX concentration/24hs plasma MTX concentration) during the first cycle predicted long-term survival. The plasma MTX concentration ratio was calculated by dividing the plasma MTX concentration at 12hr by that at 24hr during the first cycle. The plasma MTX concentration ratios were ranked as: 1) cases in which 100 < the MTX concentration ratio < 1000, and 2) those in which the MTX concentration ratio was ≤ 100 or ≥ 1000. The first rank (100 < the MTX concentration ratio < 1000) was associated with better PFS (3-year PFS: 87.5%, 95% CI:74.2%-100.0% vs 47.6%, 95% CI: 18.2%-77.7%, 5-year PFS: 83.3%, 95% CI: 68.4%-98.2%, vs 35.7%, 95% CI: 5.9%-65.5%, *P* = 0.005) and OS (3- year and 5- year OS: 91.7%, 95% CI: 83.4%- 100.0% vs 50.0%, 95% CI: 21.8%-78.2%, *P* = 0.002) than the other rank (MTX concentration ratio ≤ 100 or ≥ 1000). The survival curves based on the plasma MTX concentration ratios calculated at 12 to 24 hr during the first cycle are shown in **Figures 2C, D**.

### Prognostic Factors

In univariate analysis, the prognostic index for natural killer lymphoma–Epstein-Barr virus (PINK-E) (23) >1 and the plasma MTX concentration ratio ≤ 100 or ≥ 1000 were significant poor prognostic predictors of both PFS and OS (**Table 3**). These two factors were then included for multivariate analysis. Logistic regression was utilized for further analysis. The results showed that both PINK-E>1(*P* = 0.007 for PFS; *P* = 0.012 for OS) and the plasma MTX concentration ratios calculated at 12 to 24 hr during the first cycle ≤ 100 or ≥ 1000 (*P* = 0.040 for PFS; *P* = 0.029 for OS) were the independent risk factors in ENKTL (**Table 4**).

**Table 3:** Univariate analysis of prognostic factors.

Factors	PFS			OS		
	HR	95%CI	*P* value	HR	95%CI	*P* value
Age > 60 years old	0.981	0.125-7.681	0.985	3.336	0.669-16.626	0.141
Gender: male	0.876	0.256-2.994	0.832	1.434	0.289-7.107	0.659
ECOG PS ≥ 2	3.921	1.002-15.348	0.050	9.113	2.245-36.999	0.002
B symptoms	3.627	0.782-16.827	0.100	5.005	0.615-40.702	0.132
Ann Arbor Stage III/IV	6.436	1.862-22.251	0.003	2.905	0.584-14.452	0.193
LDH > 245 U/l	0.945	0.250-3.562	0.933	0.351	0.043-2.856	0.328
EBV DNA copy > 10^3^ copies/ml	7.896	1.009-61.818	0.049	5.086	0.625-41.368	0.128
EBV DNA copy > 0 copies/ml	39.532	0.207-7561.384	0.170	36.316	0.066-19954.117	0.264
CRP > 10 mg/l	3.622	1.054-12.441	0.041	3.018	0.720-12.649	0.131
IPI ≥ 2	6.436	1.862-22.251	0.003	2.905	0.584-14.452	0.193
PINK-E > 1	7.081	2.039-24.588	**0.002**	10.380	2.071-52.021	**0.004**
KPI > 1	2.056	0.626-6.753	0.235	2.678	0.640-11.217	0.178
The plasma MTX concentration ratio ≤ 100 or ≥ 1000	4.950	1.438-17.042	**0.011**	8.090	1.623-40.326	**0.011**

ECOG PS, Eastern Cooperative Oncology Group performance status; B symptoms consist of unexplained fever above 38℃, night sweating, or weight loss of more than 10% within 6 months; LDH, lactate dehydrogenase; EBV DNA, Epstein-Barr Virus deoxyribonucleic acid; CRP, C-reactive protein; IPI, International Prognostic Index; PINK-E, prognostic index for natural killer lymphoma–Epstein-Barr virus; KPI, Korean Prognostic Index; MTX, methotrexate.

The bold values means PINK-E>1 and the plasma MTX concentration ratio ≤ 100 or ≥ 1000 were significant poor prognostic predictors of both PFS and OS.

**Table 4:** Multivariate analysis of prognostic factors for both PFS and OS.

Factors	PFS			OS		
	HR	95%CI	*P* value	HR	95%CI	*P* value
PINK-E > 1	5.714	1.601-20.399	**0.007**	8.285	1.602-42.847	**0.012**
The plasma MTX concentration ratio ≤ 100 or ≥ 1000	3.756	1.063-13.271	**0.040**	6.211	1.208-31.925	**0.029**

PINK-E, prognostic index for natural killer lymphoma–Epstein-Barr virus; MTX, methotrexate.

The bold values means both PINK-E>1 and the plasma MTX concentration ratios calculated at 12 to 24 hr during the first cycle ≤ 100 or ≥ 1000 were the independent risk factors in ENKTL.

### The Synergistic Effect of the High-Dose MTX (HD-MTX) and Gemcitabine in the GAD-M Regimen

**Figure 3:**
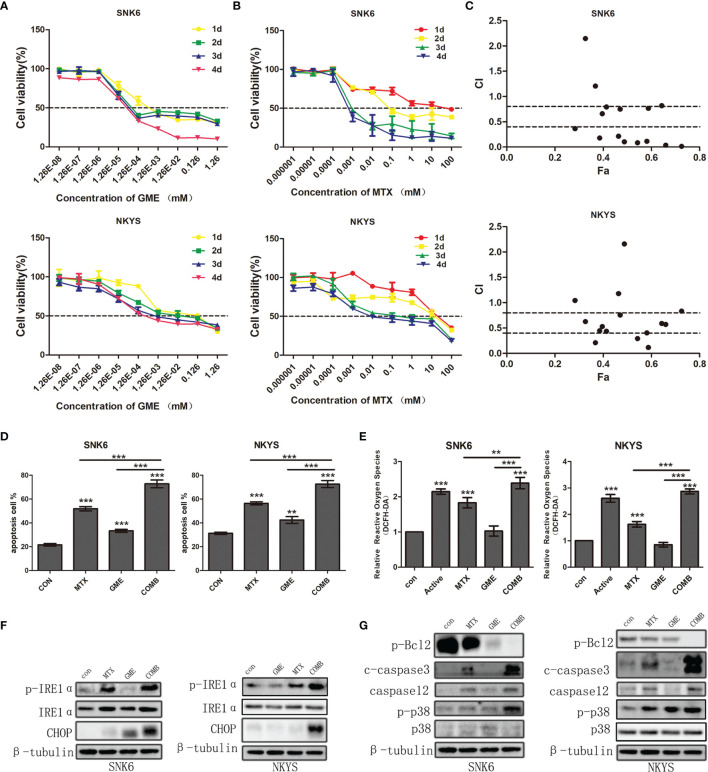
The synergistic effects of HD-MTX and gemcitabine-induced NK/T-cell lymphoma cell apoptosis through ER stress. **(A, B)** the effects on NK/T-cell lymphoma cell growth of MTX or gemcitabine. **(C)** the synergistic effects of HD-MTX combined to gemcitabine calculated using CalcuSyn. **(D)** cell apoptosis was performed followed by flow cytometric analysis. **(E)** intracellular ROS was measured by the MFI of DCFH-DA. **(F, G)** the ER stress and apoptosis-related markers were detected by western blotting. ** means P < 0.01, *** means P < 0.001.

The IC50 values of gemcitabine on SNK6 and NKYS ranged between 43.95 nM~ 2.646 μM and 2.799 μM~ 40.63 μM, respectively, after treatment with SNK6 and NKYS for 1-4 d (**Figure 3A**). The IC50 values of MTX ranged between 1.328 μM~ 20.43 mM and 72.42 μM~ 21.51 mM, respectively (**Figure 3B**). Compared to NKYS, SNK6 was more sensitive to MTX and gemcitabine. HD-MTX combined with gemcitabine had a synergistic killing effect on NK/T-cell lymphoma (**Figure 3C**).

We next sought to understand the mechanisms underlying the synergistic effect of MTX and gemcitabine. The percentage of Annexin V ^+^ (a marker of apoptosis) and PI ^+^Annexin V ^+^ (markers of cell death) cells was found to increase after treatment with MTX and gemcitabine respectively, and showed marked upregulation in the combination group (**Figure 3D**). In addition, the production of intracellular reactive oxygen species (ROS) was increased in MTX, and the drug combination-treated cells (**Figure 3E**). Considering an excess of ROS would induce cell apoptosis, we hypothesized that cell apoptosis could be promoted by endoplasmic reticulum (ER) stress activated by the severe release of ROS.

To validate this hypothesis, we next detected the change in ER stress sensor. We found that the expression of p-IRE1α increased in the MTX group and was much higher in the combination group. Moreover, the accompanying upregulation of C/EBP homologous protein (CHOP) indicated the activation of ER stress (**Figure 3F**).

In order to identify that cell apoptosis was the result of ER stress, we subsequently detected the expression of p-p38 and Caspase12. We found that these two drugs combination augmented cell apoptosis. P-p38 inhibited the expression of Bcl2 protein, and increased the amount of cleaved caspase3. Moreover, an accumulation of CHOP and caspase12 was also found and contributed to severe cell apoptosis (**Figure 3G**). Taken together, our results supported the notion that MTX and gemcitabine combination could extremely elevate intercellular ROS which in turn induced NK/T-cell lymphoma cell apoptosis by activating ER stress.

## Discussion

This study first investigated the effect of the GAD-M regimen on treatment outcomes in newly diagnosed ENKTL. Our results indicated that GAD-M chemotherapy was indeed effective for the treatment of newly diagnosed ENKTL. The ORRs after 2 and 6 cycles of GAD-M were 94.4% (95% CI: 87.0%-100.0%) and 91.6% (95% CI: 82.6%-100.0%), respectively, with both clearly exceeding the expected ORR of 80% and the threshold ORR of 60% (21, 22). Moreover, these results were similar to the highest ORRs achieved by the PGEMOX/GELOX and GGDP regimens in recent reports (24, 25). The GAD-M regimen had a 100% ORR and a 90.3% CRR after 6 cycles when sandwiched with IFRT and was therefore effective for stage I/II ENKTL patients. The results provide a rationale for further randomized controlled trials.

MTX, an antimetabolite drug derived from folic acid, is unaffected by the multidrug resistance pathway and may have a synergistic effect with either L-asparaginase or peg-asparaginase when used in acute lymphoblastic leukemia (26–28). It is a component of regimens that have been shown to be effective in relapsed/refractory or stage IV ENKTL, such as SMILE and AspaMetDex (12, 13, 16). It was previously reported that MTX combined with irradiation could inhibit NF-κB activation in ENKTL cells (29). Therefore, MTX is an important drug in newly diagnosed ENKTL. However, no other study has explored the use of this drug combined with both peg-asparaginase and gemcitabine in ENKTL. Our study is the first study to confirm the effectiveness and safety of the GAD-M regimen in ENKTL. Meanwhile, the MTX is rather important for CNS prophylaxis in lymphoma (30), while the history data indicated that the 2-year and 5-year CNS relapse rates were 5.1% and 7.2% in ENKTL (31). Hyera Kim et al. found the tendency of reducing the cumulative incidence of CNS relapse in the high-risk CNS-PINK group with intermediate-dose (ID-MTX) (32). In our study, there was no CNS relapsed with the GAD-M regimen during the follow-up. Based on the recent study and our findings, we hypothesized that the combination of GAD-M with MTX might have a potential influence on CNS prophylaxis in ENKTL.

Moreover, we found that the MTX concentration ratio had a prognostic value in ENKTL. Pearson et al. showed that neither plasma MTX concentration nor MTX clearance predicted survival in childhood acute lymphoblastic leukemia (33). However, our results indicated that the MTX concentration ratio but not the plasma MTX concentration predicted the effect of the GAD-M regimen and survival in ENKTL. Hence, the clinical data presented in this study may indicate that appropriate MTX clearance (neither too high nor too low) could predict a good outcome in ENKTL. However, one limitation of our study was that the mechanism of action of MTX clearance was still unknown. It would be interesting to understand this issue and warrants further investigation.

For stage I/II disease, after the initial 2 cycles of GAD-M, the observed CRR was 54.8%. Upon completion of sandwiched IFRT and a subsequent 4 cycles of GAD-M, the CRR increased to 90.3%. This indicated that sandwiched IFRT could increase the CRR of GAD-M. Furthermore, we noticed that most disease progressions occurred within 18 months and there were very few cases (only two cases) of new relapse or progression after two years of follow-up. This indicated that the response to GAD-M sandwiched with IFRT could be durable in early-stage ENKTL. The study data support further development of GAD-M for early-stage ENKTL however its utility for advanced stage ENKTL is unclear because of the limited number of advanced-stage patients in this study.

The most common toxicities observed in our study were hypoalbuminemia, anemia, and leukocytopenia during the GAD-M treatment. We noticed that all patients in our study suffered from hypoalbuminemia and this may have been caused by peg-asparaginase. This adverse reaction was usually graded as 1/2 (94.4%) and was reversed by albumin infusion. The rate of grade 3/4 neutropenia (36.1%) in GAD-M was much lower than that observed in SMILE (12) and similar to that observed in PGEMOX/GELOX studies (17). Grade 3/4 thrombocytopenia (16.7%) was less frequent than has been observed for other regimens (12, 17). However, anemia should be carefully monitored both during and after GAD-M chemotherapy. Only one elderly patient died of electrolyte disorders caused by severe vomiting. This treatment toxicity was similar to that reported in the SMILE study (12, 34). Other non-hematological adverse events were mostly graded as 1/2 and were transient and manageable.

Based on the history data and our study (12, 17, 31, 32), the GAD-M regimen may show more effect on CNS prophylaxis than the PGEMOX/GELOX regimens due to the MTX, and better tolerance than the SMILE for ENKTL.

In addition, to understand the mechanism of MTX combined with gemcitabine in the treatment of NK/T-cell lymphoma, we did rational research. Depending on our results, we found that the synergistic killing effect on NK/T-cell lymphoma cells was induced grievous apoptosis by MTX and gemcitabine through the ER stress-dependent pathway. In this study, we explored the molecular mechanism of MTX combined with gemcitabine in killing NK/T-cell lymphoma cells, and provides a theoretical basis for clinical use of GAD-M regimen in the treatment of ENKTL patients.

Although the GAD-M regimen was effective in ENKTL, there were some limitations worth mentioning. First, this was a single-center phase II study, and phase III multicenter randomized control trial is needed to confirm the findings of this study. Second, the effect of GAD-M on the advanced stage might have caused some bias in the assessment of treatment response due to the limited number of advanced ENKTL in this study. Third, the influence of the MTX concentration ratio was an explorative analysis in the phase II study, the impact of MTX clearance in ENKTL still needs to future explored.

In conclusion, we demonstrated that the GAD-M regimen could provide a high ORR in patients with newly diagnosed ENKTL, especially for those with stage I/II disease. IFRT could improve the CR rate in ENKTL. GAD-M combined with IFRT in stage I/II ENKTL was feasible and well-tolerated. The MTX concentration ratio could predict patients’ outcomes in this group and might be an interim assessment index for ENKTL. Furthermore, our rational research results supported the synergistic effect of the HD-MTX and gemcitabine in NK/T cell lymphoma cell lines. The GAD-M regimen may be a new choice for first-line treatment in ENKTL, especially in stage I/II disease.

## Data Availability Statement

The datasets generated for this study are available on request to the corresponding authors.

## Ethics Statement

The study involving human participants was reviewed and approved by the ethics committee of the Sun Yat-sen University Cancer Center. This study’s protocol was complied with the Declaration of Helsinki. The patients provided their written informed consent to participate in this study.

## Author Contributions

YW, CQW, PS contributed equally to this article. YW, PS, WQJ, JJH and ZML contributed to study design and drafting of the manuscript. YW, CQW, PS, PPL, HY, HYW, HLR, SL, WQJ and ZML recruited patients for this study. YW, PPL and HY collected data. YW, CQW and PS did the analyses. All the authors were involved in the provision of study materials and patients, and data interpretation. All authors gave final approval to submit for publication.

## Funding

This work was supported by grants from National Natural Science Foundation of China (nos.82104273, nos.82103579, nos.82073917, nos.81872902), National Science and Technology Major Project (nos. 2018ZX09734003), Guangdong Medical Research Foundation (nos. A2020145) and the Sun Yat-sen University Cancer Center Clinical Research 308 Program (nos. 2014-fxy-106 and 2016-fxy-079).

## Conflict of Interest

The authors declare that the research was conducted in the absence of any commercial or financial relationships that could be construed as a potential conflict of interest.

## Publisher’s Note

All claims expressed in this article are solely those of the authors and do not necessarily represent those of their affiliated organizations, or those of the publisher, the editors and the reviewers. Any product that may be evaluated in this article, or claim that may be made by its manufacturer, is not guaranteed or endorsed by the publisher.
